# A case study in photosynthetic parameters of perennial plants growing in natural conditions

**DOI:** 10.1186/s12870-025-07133-1

**Published:** 2025-08-08

**Authors:** Agata Dziwulska-Hunek, Beata Myśliwa-Kurdziel, Arkadiusz Matwijczuk, Mariusz Szymanek

**Affiliations:** 1 Department of Biophysics, University of Life Sciences in Lublin, Akademicka 13, Lublin, 20-950 Poland; 2 Faculty of Biochemistry, Biophysics and Biotechnology, Jagiellonian University, Gronostajowa 7, Kraków, 30-387 Poland; 3 ECOTECH−COMPLEX– Analytical and Programme Centre for Advanced Environmentally−Friendly Technologies, Maria Curie−Skłodowska University, Lublin, Poland; 4 Department of Agricultural, Forest and Transport Machinery, University of Life Sciences in Lublin, Głęboka 28, Lublin, 20-612 Poland

**Keywords:** Photosynthetic efficiency, Electron transport, Fluorescence lifetime, Photosynthetic pigments, Legume plants

## Abstract

The research objective of this study was to determine the physiological condition of selected legume species growing under natural conditions, based on analysis in them: photosynthetic efficiency, chlorophyll fluorescence lifetime and photosynthetic pigment content. Evaluation of these parameters allows early detection of stress factors affecting the physiology of the plants and the determination of their adaptive capacity. The plants selected for the study included: white clover, red clover, alfalfa, and common sainfoin. The presented results revealed that the photosynthetic activity was lower in red clover but higher in common sainfoin. Longer τ_1_ and τ _2_ fluorescence lifetimes of Chl *a* were measured in red clover and common sainfoin, while shorter lifetimes were recorded for white clover and alfalfa. Shorter lifetimes suggest greater activity of other chlorophyll excitation pathways, including in particular ones related to energy dissipation in the photosynthetic antennae. The content of chlorophyll *a*,* a + b* was noticeably higher in the leaves of red clover, and lower in the leaves of common sainfoin. The varied values of photosynthetic parameters measured in the analyzed perennial plants from the same *Fabaceae* family indicate the ease with which they adapt to environmental conditions. The studied plants are excellent sources of nitrogen and fodder protein, rendering them interesting in the context of sustainable agriculture.

**Graphical abstract:**

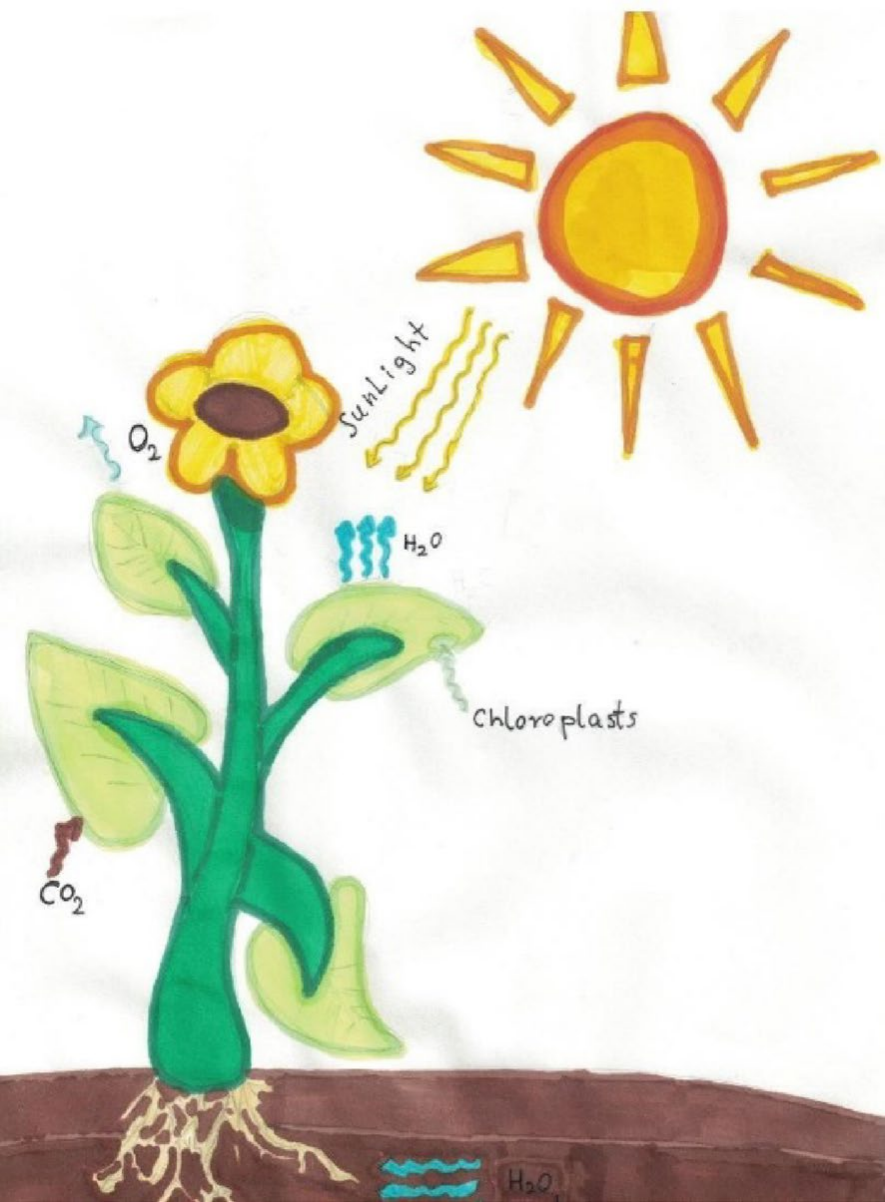

**Supplementary Information:**

The online version contains supplementary material available at 10.1186/s12870-025-07133-1.

## Introduction

There is currently a growing interest in the development of sustainable agriculture aimed at minimizing the negative impact of environmental conditions and conserving natural resources. Moreover, the importance of ensuring economic viability of present and future agriculture has never been greater [1].

The Fabaceae, also known as legumes, are divided into two primary groups: thick-seeded, e.g. lupin (*Lupinus* L.), pea (*Pisum sativum*), broad bean (*Vicia faba)*, andsoybean (*Glycine max*); and small-seeded such as alfalfa (*Medicago*), clover (*Trifolium*), sainfoin (*Onobrychis*), and bird’s-foot trefoil (*Lotus corniculatus*). Legume production is particularly important in the context of sustainable agriculture, especially given the current climatic changes affecting Europe as well as other continents [2, 3]. Perennial plants find a wide variety of applications, including in terms of the ecological benefits related to their contribution to the condition of soils and processes taking place therein– they are efficient in reducing environmental pollution through symbiosis with rhizobia, which allows them to bind atmospheric nitrogen and consequently reduce the need for artificial fertilization [4]. Moreover, they are an excellent choice as pioneer crops and are often used in organic farming as additional enhancement of soil mineral content that, again, allows farmers to reduce the need for synthetic fertilization. The cultivation of catch crops facilitates temporary extraction of soil nutrients that would otherwise be washed away. Secondly, one should also consider the economic aspect– the plants are a good source of high-protein fodder and some, e.g. alfalfa or clover, are also used in the production of human food as valuable sources of sprouts. Furthermore, perennial legumes are highly pollinator-friendly due to their good honey output; they also have various medicinal benefits [5].

**Fig. 1:**
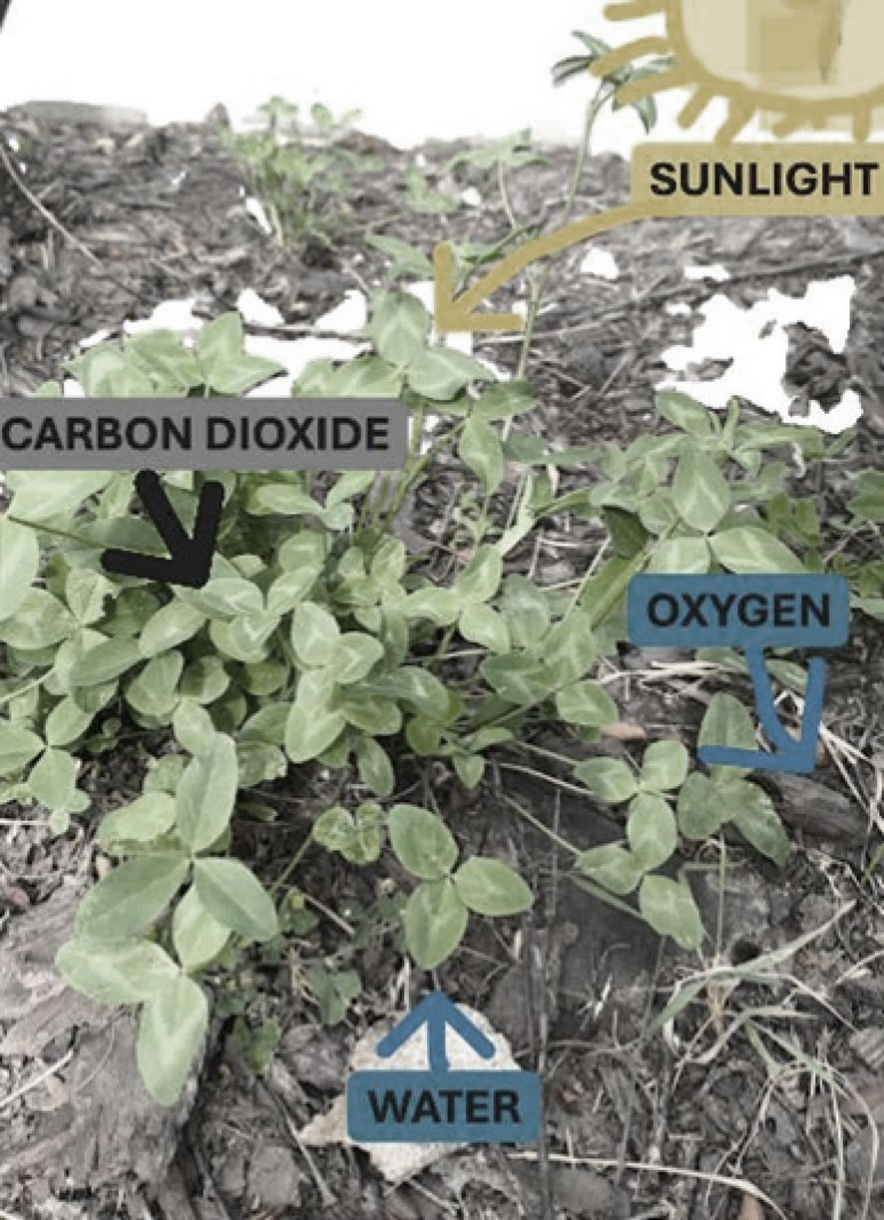
The process of photosynthesis - performed by Wiktoria Hunek

Life on Earth is indisputably dependent on the phenomenon of photosynthesis (Fig. 1.) [6]. The process takes place in chloroplasts and entails a conversion of carbon dioxide and water into glucose and oxygen using sunlight energy. Sunlight energy reaches the leaves and is absorbed by photosynthetic pigments located in the photosynthetic complexes of thylakoid membranes in chloroplasts. The absorbed energy triggers photosynthetic electron transport, which facilitates the biosynthesis of assimilates (ATP and NAPDH) necessary for the production of plant biomass. Any excess energy is dissipated [7].

Different types of plants are exposed in their natural habitats to numerous and varying environmental conditions that may hinder their physiological processes [8]. Plant yields can change due to environmental stress resulting from climate changes [9], which in turn may impact the food production and food security [10]. The process of photosynthesis is the primary driving force behind the growth and production of plant biomass [11, 12]. With the use of the most modern, practically non-invasive and quick fluorescence methods, it is now possible to control the general condition of plants during their growth and cultivation [13, 14].

The research problem explored in our study pertained to a real-time comparative analysis of photosynthetic parameters obtained from in vivo measurements of chlorophyll fluorescence in selected species of perennial in natural conditions legumes growing in the same area, specifically white and red clover, alfalfa, and common sainfoin. Supplementary measurements included the content of photosynthetic pigments (chlorophylls *a*,* b* and carotenoids) in the leaves of the plants as well as mean fluorescence lifetimes measured for chlorophyll present in the leaves. Such measurements allow one to determine plants’ overall physiological condition and facilitate timely prevention of the negative impact of adverse environmental conditions on plants growing in natural conditions. One-way analysis of variance (ANOVA) was used for the variables analyzed to assess the significance of differences between the perennial faba bean plant species studied. The null hypothesis (H₀) was formulated, assuming that there were no significant differences in mean in vivo chlorophyll fluorescence values, chlorophyll and carotenoid contents and fluorescence lifetimes between species growing under natural conditions in the same study area. The alternative hypothesis (H₁) was that at least one of the analyzed variables differed significantly between the study species.

The obtained results are relevant to eco-physiological studies on ecosystems threatened by phytotoxic factors and aimed at determining the nutritional requirements of crops.

## Materials and methods

### Plant material

As an research material were used leaves gathered from four chosen species white clover (*Trifolium repens* L.), red clover (*Trifolium pratense* L.), alfalfa (*Medicago sativa* L.), and common sainfoin (*Onobrychis viciifolia Scop.*). which are typical Fabaceae species for natural ecosystem experimental field of University of Life Sciences in Lublin (51°13′21.9″ N, 22°37′55.85″ E). The plants studied were 2 years old and grew in an unshaded area. Leaves were collected on the morning of 21 April 2016 at the rosette leaf stage (scale BBCH 19).

The experimental and field studies on plants used in the research, including the collection of plant material, comply with relevant institutional and national guidelines and legislation effective at the Research Centre for Cultivar Testing [15].

### Photosynthetic efficiency

Photosynthetic efficiency was measured based on in vivo chlorophyll fluorescence using a Mini-PAM 2000 Photosynthesis Field analyzer (Waltz, Germany). The instrument measures modulated fluorescence, primarily in the PSII photosystem. The saturating pulse intensity was 18 000 µmol m^− 2^s^− 1^ PAR and the actinic light was 6 000 µmol m^− 2^s^− 1^ PAR. The plants were in light adapted state. An analysis of the results was conducted using WinControl Software for PAM Fluorometers (Waltz, Germany). The following parameters were calculated based on the measured chlorophyll fluorescence: the effective quantum yield (Y (II)) of the photochemical energy conversion at PS II reaction centers and the electron transport rate (ETR) [16] according to Eqs. 1 and 2, respectively.

1 $${\text{Y }}\left( {{\text{II}}} \right){\text{ }}={\text{ }}({\text{Fm}}^{\prime }-{\text{F}})/{\text{ Fm}}^{\prime }$$

where: F– fluorescence intensity measured in light adapted state before the saturating pulse, and Fm’– fluorescence intensity reached during the saturating pulse in light adapted state

2 $${\text{ETR}}\,=\,{\text{Y }}\left( {{\text{II}}} \right){\text{ }}*{\text{ PAR }}*0.{\text{5 }}*{\text{ }}0.{\text{84}}$$

where: Y (II)– the effective quantum yield, PAR– photosynthetically active radiation; the factor 0.5 accounts for the fact that roughly 50% of all absorbed quanta reach PS II, the standard factor 0.84 corresponds to the fraction of incident light absorbed by the leaf. The measurement was taken at the top of the plant, i.e. its topmost leaves, to account for the best light absorption. All measurements were done in ten replicates for each species studied.

Following the procedure described earlier, an additional measurement of the photosynthetic efficiency of selected perennial plant species was carried out on the morning of 25 May 2016. At the time of measurement, the plants were at the main shoot growth stage, corresponding to the BBCH 34 stage.

**Fig. 2:**
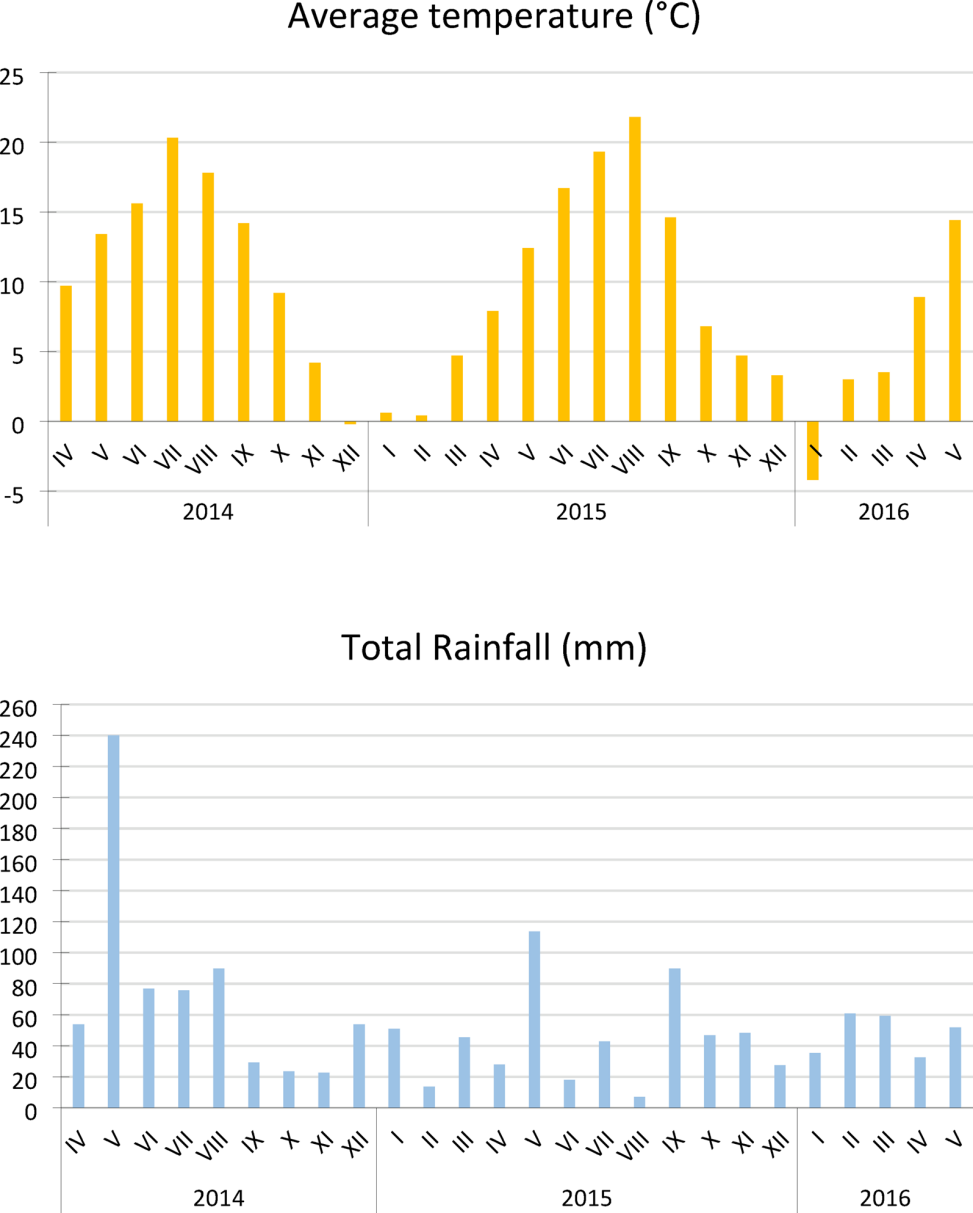
The meteorological conditions observed during the growing period of selected Fabaceae plants species (**A**) average temperature and (**B**) total rainfall

The meteorological conditions observed during the growing period of selected Fabaceae plants species (Fig. 2.) were described on the basis of measurement and observation data from the meteorological station in Lublin [17].

### Fluorescence lifetimes

Measurements of fluorescence decay were taken using a K2 phase-modulation fluorimeter (ISS, USA) [18–20]. The leaves were crushed in a mortar in Hepes–NaOH buffer (25 mM; pH 7.8) containing 1 mM of MgCl_2_, 1 mM of EDTA, and 0.4 M of sorbitol. The homogenate was diluted using the buffer so that the absorbance of the sample at 440 nm did not exceed 0.15. The sample was excited with 440 nm waves and the fluorescence was observed through a KV550 cut-off filter (λ > 550 nM). Measurements were done in a 1 × 1 cm cuvette, at room temperature, at 10 frequencies of exciting wave modulation (in the range of 2–200 MHz), relative to the diffusing suspension (Ludox, Sigma Aldrich). Vinci2 software (ISS, USA) was used in the analysis for a double exponential and triple exponential fluorescence decay model to determine which best corresponded to the results obtained. However, the third component, if detected, had very long values and tiny fractional intensity, and was therefore not taken into consideration. Average fluorescence lifetime was calculated as < τ_i_ > = Σ_i_ τ_i_ × f_i_. Leaf samples for analysis were collected by hand, selecting the uppermost leaves in the rosette of the plant that had the best light absorption capacity. All measurements were taken in five replications for each species studied.

### Photosynthetic pigments

Determination of the content of photosynthetic pigments. Photosynthetic pigments were isolated from leaves using acetone containing 0.01% w/v BHT (butylated hydroxytoluene), in darkness, after which the content of chlorophyll and carotenoids, respectively, was determined. The UV–Vis spectrum was measured using a Carry Bio 300 spectrometer (Agilent Technologies, CA, USA) and analyzed in accordance with the procedure published by Lichtenthaler and Buschmann [21]. Leaf samples for analysis were collected by hand, selecting the uppermost leaves in the rosette of the plant that had the best light absorption capacity. All measurements were taken in three replications for each species studied.

### Statistical analysis

The experimental results were processed with STATISTICA 13.1 software, using the ANOVA variance analysis and post-hoc LSD test at the significance level of α < 0.05. The correlations between variables were determined using Pearson’s linear correlation coefficient.

## Results and discussion

### Photosynthetic efficiency

It is worth noting at the outset that the perennial legume species analyzed were two years old at the time of the leaf analysis, in April 2016. During this period, the average temperature was 8.9 °C and total precipitation was 32.6 mm. April of the sowing year (2014) had higher values of both mean temperature and total rainfall compared to 2015 and 2016 (Fig. 2). The low temperature and limited rainfall could certainly have contributed to the reduced photosynthetic efficiency in the plants studied. After a month (May 2016), the average temperature increased to 14.4 °C and total precipitation was 51.9 mm. May 2016 was slightly warmer and precipitation lower compared to 2014 and 2015 (Fig. 2.). The photosynthetic capacity of the selected perennial plants increased significantly - in the case of red clover even twice. This increase may have been influenced by both the higher temperature and the advanced stage of plant development.

Table 1. shows the results of photosynthetic parameter measurements for the respective species of plants from the Fabaceae family. Compared to the other plants, alfalfa showed overall the highest values of photosynthetic parameters measured in light adapted leaves. The photosynthetic efficiency Y (II) measured for white clover, alfalfa and sainfoin was similar and remained within the range from 0.443 (white clover) to 0.468 (common sainfoin). The photosynthetic yield was the highest for sainfoin, nearly twice as high as in the case of red clover (0.274). The low value of photosynthetic efficiency observed for red clover indicates that the light to which it was adapted, i.e. 1015.70 µmol m^− 2^ s^− 1^, may have been too intensive for the plant, which triggered the process of excess energy dissipation. After one month, the photosynthesis index value increased and ranged from 0.521 (red clover) to 0.697 (sainfoin) (Table S1.). In the case of red clover, an almost twofold increase was observed compared to the earlier measurement. Plants growing in temperate climate zones are often exposed to both low and high temperature stress. In most species, at full development and under non-stress conditions, the maximum value of the PSII photochemical efficiency (Fv / Fm) reaches a value of about 0.83. A decrease in this parameter indicates that the plant under study has been influenced by stress factors such as too high or too low a temperature, water deficiency or excess, soil salinity or the presence of heavy metals. Radiation in the photosynthetically active PAR range plays a key role in photosynthesis and plant development [13, 22]. During the day, the quantity and quality of photosynthetically active radiation can vary considerably. At low light intensities, most of the absorbed energy can be efficiently used in photosynthesis, whereas at high PAR intensities only a small part is used [23]. An excess supply of PAR to the antenna complexes triggers a protective mechanism of the photosynthetic apparatus, especially PSII. Electron transport is slowed down and leads to partial degradation of a key protein. Stress induced by too low (100 µmol photons m^− 2^ s^− 1^) or too high PAR intensity (1500 µmol photons m^− 2^ s^− 1^) in growing barley seedlings did not cause significant changes in the Fv/Fm parameter 24 h after the onset of the stress factor. Changes were noticed after 7 days of stress [13]. Notably, its electron transport rate (ETR) was only slightly lower than that measured in alfalfa, despite the latter showing much higher Y(II) after having been adapted to light of similar intensity.

In our study, the effective quantum yield fluctuated between 0.274 (red clover) and 0.468 (common sainfoin), and the electron transport rate between 27.33 and 110.12 µmol electrons m⁻² s⁻¹ for white clover and alfalfa, respectively. When analyzing data collected for white clover and sainfoin, for which relatively low ETR values were observed, one should note that in the case of these particular plants, light intensity may have been a factor limiting the rate of electron transport.

The discrepancies in terms of photosynthetic efficiency provide information about the actual physiological condition of the analyzed plants due to various environmental factors, including intensity of light [24].

**Table 1:** Photosynthetic parameters measured for perennial leguminous plants

Plant species	PAR (µmol m^− 2^ s^− 1^)	TEMP. (^o^C)	F	Fm^’^	Y (II)	ETR (µmol electrons m⁻² s⁻¹)
White clover	184.50^b^ ± 66.22	10.56^b^ ± 0.25	364.20^ab^ ± 26.46	699.40^ab^ ± 88.10	0.443^a^ ± 0.042	27.23^b^ ± 8.62
Red clover	1015.70^a^ ± 163.98	11.48^a^ ± 0.21	496.10^a^ ± 89.23	655.20^b^ ± 110.58	0.274^b^ ± 0.031	96.27^ac^ ± 10.11
Alfalfa	1035.40^a^ ± 382.92	10.04^b^ ± 0.16	508.80^a^ ± 69.15	1204.00^a^ ± 323.44	0.452^a^ ± 0.075	110.12^a^ ± 18.78
Common sainfoin	347.40^b^ ± 138.24	10.40^b^ ± 0.16	221.00^b^ ± 47.84	478.70^b^ ± 139.99	0.468^a^ ± 0.055	59.36^bc^ ± 11.92

Fisher Test (LSD) α < 0.05, ± standard deviation, a–c– different letters indicate statistically significant differences between the plants, *n* = 10

F– fluorescence intensity, measured in light adapted state before the saturating pulse, Fm’– fluorescence intensity reached during the saturating pulse in light adapted state, PAR– photosynthetically active radiation, ETR– relative electron transport rate, Y (II)– effective quantum yield of photochemical energy conversion at PS II reaction centers, TEMP.– temperature of the leaf during measurement

Staniak and Baca [8] observed that the quantum yield of photosystem II in the leaves depended mostly on the plant species. The value was significantly higher for alfalfa and red clover when compared to white clover. This indicated higher photosynthetic efficiency (PII) and generally higher vitality of the two species. In our study, the highest values of photosynthetic efficiency were observed for sainfoin, alfalfa, and white clover, while the lowest Y (II) value was recorded for red clover. The photosynthetic quantum yield value suggested better overall condition of the former three species. In other studies conducted by Ćwintal and Olszewski [25], it was observed that the fodder value of alfalfa was the highest during the budding phase. As the plants matured from the budding to the flowering phase, their content of protein decreased, and the content of raw fiber increased. The study indicated that stimulation with laser light had no significant impact on the intensity of photosynthesis, but crop harvest during plant vegetation did. During the first crop harvest, the highest photosynthetic efficiency was recorded, considerably higher than for the 2nd and 3rd after-crop [25].

It has been reported that legumes are very sensitive to reduced sunlight access. The efficiency of their photosynthetic system decreases in plants growing in the shade [26]. As follows from literature data, fluctuations of chlorophyll *a* fluorescence parameters recorded for rice plants at varying light intensities were related to the size of PSII antennae [27]. Harvests worldwide are negatively affected by draughts, which undoubtedly relates to the current climate changes [28]. At the same time, elevated nitrogen content under salt stress conditions increased photosynthetic activity by 44% in the fodder legume *Sulla carnosa*, while without salination the result reached 57% [29]. In our study, the photosynthetic efficiency recorded for common sainfoin was 71% higher than in red clover. The increased photosynthetic parameters observed for sainfoin were due to the favorable conditions present in the local ecosystem.

In other studies, it has been reported that leaf damage caused by herbivorous insects had an impact on the gas exchange reaction. As follows from the research hypothesis posed in the study, damage to leaves reduced photosynthetic activity [30].

The rate of electron transport in photosynthesis determines the effectiveness with which the products of light-mediated photosynthetic reactions, NADPH and ATP, are substituted, which in turn determines the rate of biomass gain. When growing in natural conditions, plants experience constant changes in the quality and quantity of sunlight received and regulate their photosynthetic electron transport systems accordingly [31]. The electron transport rate grows with increasing light intensity, until it reaches the point of saturation and photoinhibition. The curve representing this process is characteristic for each particular plant species [32]– [33].

**Fig. 3:**
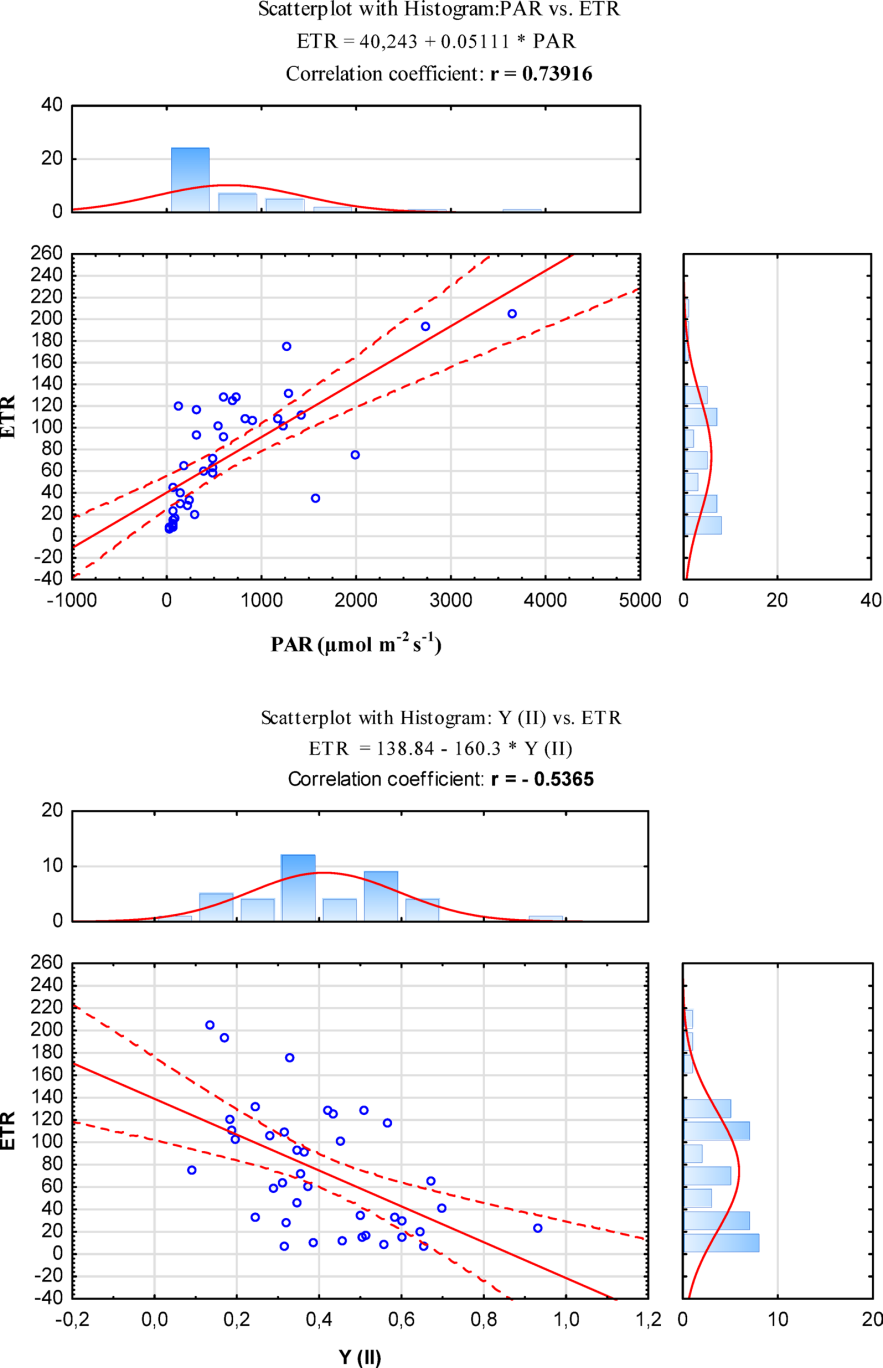
Scattering plot for all the plant species studied with a histogram showing correlations between: (**A**) photosynthetically active radiation (PAR) and relative electron transport rate (ETR); (**B)** quantum efficiency of the photochemical reaction Y(II) and relative electron transport rate (ETR). The bold value of the correlation coefficient r indicates a significant statistical difference, at a significance level of α < 0.05

In our study pertaining to plants growing at the same location, next to each other, we observed a strong positive correlation between photosynthetically active radiation (PAR) and electron transport rate (ETR) (Fig. 3A. and S3A.). It is worth noting that the positive correlation between the study variables was statistically significant (α < 0.05) and indicated the direction of their mutual relationship, i.e. an increase in one variable was simultaneously associated with an increase in the other. Lower PAR intensity correlated with lower electron transport, as could be observed for white clover and common sainfoin. Conversely, when the PAR ratio increased, so did ETR– in our case, this was seen in red clover and alfalfa (Table 1.). Over the course of a day, the intensity of light can fluctuate between 0 and 2000 µmol m^− 2^ s^− 1^ due to the cloud cover or changing position of the sun relative to shade-giving objects [34]. Undoubtedly, changing insolation and other environmental conditions will affect photosynthetic efficiency by e.g. triggering regulation of electron transport pathways [35]. In turn, the correlation observed between ETR and Y(II) turned out to be moderately negative (Fig. 3B. and S3B.), which suggests that each of the plant species reacted to light somewhat differently. It should be noted that the negative correlation between the study variables showed statistically significant differences (α < 0.05), indicating the opposite direction of their variability. Perennial legumes are photophilic plants that prefer high insolation and relatively high temperatures. The spectrum of photosynthetic activity is closely related to the spectrum of chlorophyll absorption [36].

### Fluorescence lifetimes

The results of fluorescence lifetime measurements for chlorophyll *a* present in the leaves of the analyzed plant species are presented in Table 2. Two components of the fluorescence decay time (τ_1_ and τ_2_) were identified in the analyzed samples, with relative share of f_1_ and f_2_, respectively. Fluorescence lifetimes measured in the leaves of the respective plant species were between 0.293 ns and 0.594 ns for the τ_1_ component, and between 2.46 ns and 3.19 ns for the t_2_ component. The share of respective components was between 22% and 82% (f_1_), and between 18% and 78% (f_2_). The mean fluorescence lifetime was the longest for red clover, followed by white clover, and the lowest (and similar) for sainfoin and alfalfa. A shorter fluorescence lifetime indicates more efficient energy transfer from the antennae, and consequently higher photosynthetic efficiency.

In another study, fluorescence lifetime measurements in black salsify leaves returned values between 0.09ns and 2.60 (τ_1_) with the relative share of 16–100%, and between 0.77ns and 2.77ns (τ_2_) with the share of between 22% and 84%. It was observed that (τ_1_) lifetimes in leaves grown from 2012 seeds were above 1ns in all samples examined. In plants grown from 1-year-old seeds, fluorescence lifetimes were generally longer as compared to plants grown from 4-year-old seeds (harvested in 2009), with the latter mostly yielding results under 1ns, the exception being seeds subjected to stimulation with He-Ne laser light with 10-minute exposure nasion [37]. In our present study, the average lifetime was under 3.00ns. Shorter fluorescence lifetimes indicate higher efficiency of other chlorophyll excitation channels, which is partially related to energy dissipation in the photosynthetic antennae. The prevalent interpretation in literature is that short fluorescence lifetimes (under 3.00 ns) are a sign of plant ageing [38, 39]. It is likely that the obtained lifetime results were affected by stress during growth and development stages in the lives of the perennial plants.

Some data available in literature suggest that for a typical leaf, the average fluorescence lifetime is approximately 290ps [40]. The mean lifetimes measured for wheat and corn was 1ns, while lifetimes decreased to 0.45ns were reported in corn experiencing water shortages [41].

In a study conducted by Lei et al. [42], lifetimes measured in tobacco leaves infected with a virus were shorter as compared to healthy leaves.

**Table 2:** Fluorescence lifetimes in homogenates from leaves of perennial leguminous plants

Plant species	τ_1_ (ns)	f_1_	τ _2_ (ns)	f_2_	$\:<\varvec{\uptau\:}>$ (ns)
White Clover	0.318 ± 0.02	0.462	2.81 ± 0.06	0.519	1.605 ± 0.012
Red Clover	0.594 ± 0.02	0.217	2.81 ± 0.03	0.783	2.329 ± 0.007
Alfalfa	0.293 ± 0.01	0.675	2.46 ± 0.08	0.255	0.825 ± 0.009
Common sainfoin	0.323 ± 0.02	0.817	3.19 ± 0.70	0.183	0.846 ± 0.144

τ_1_, τ _2_– lifetime components, f_1_, f_2_– lifetime share, $\:\stackrel{\sim}{\tau\:}$– mean lifetime, ± standard deviation, *n* = 5

### Photosynthetic pigments

Light plays a key role in photosynthesis [43], while chlorophyll determines the intensity of photosynthesis by acting as the main pigment responsible for absorbing light energy in the visible wavelength range (λ = 400–700 nm). Chlorophyll content is an important indicator of the physiological state of plants and their ability to carry out photosynthesis [44]. Chlorophyll levels and overall plant development and yield are significantly influenced by weather conditions, plant age, and morphological and physiological characteristics of individual species and cultivars [45–47]. Significant anatomical differences have been observed among the legume species tested, including a more irregular epidermal cell structure and larger size of stomata in perennial vetch compared to lucerne and sainfoin [48]. On the other hand, a study by Cooper and Qualls [49] showed that changes in leaf morphology and chlorophyll content in legumes under shade conditions were associated with an increase in the ratio of leaf area to leaf mass. Lucerne and clover grown in full light showed a higher number of stomata per unit leaf area than the same species developing in shade. In addition, leaves developing under sunlight conditions were thicker than those produced under light-limited conditions.

Reduced chlorophyll levels in leaves are often associated with environmental stress and plant ageing processes [50]. Prolonged drought can lead to a significant decrease in chlorophyll content, which negatively affects plant growth and development, as has been shown for alfalfa [51].

Table 3. presents the measurements of photosynthetic pigment content in the leaves of the respective legumes selected for this study. The content of chlorophyll a ranged from 159.94 µg g^− 1^ (sainfoin) to 389.86 µg g^− 1^ (white clover), and of carotenoids from 56.94 µg g^− 1^ (white clover) to 157.21 µg g^− 1^ (alfalfa). In a study conducted by Ćwintal et al. [46], the measurements of chlorophyll *a*, *b* and carotenoid content revealed more of the same (the pigments) in young plants (1–2 years old) as compared to older plants (5–6 years old). In another study, the content of chlorophyll *a + b* in the leaves of black salsify was higher in plants grown from seeds harvested in 2012 as compared to plants grown from 2009 to 2010 seeds [37].

Fernández-Marín et al. [52] conducted a study on the fluctuations of carotenoid content in respective stages of vegetation: sprouting and seedling development, in wild leguminous plants (*Caesalpinioideae*, *Miomosoideae*, *Papolionoideae* and other *Fabaceae*). The content of carotenoids increased slightly in the sprouting period for one subfamily Papilionoideae. Simultaneously, the content of chlorophylls of the same subfamily also grew rapidly as the leaves started to transform into photosynthetic organs. In our study, it was observed that the content of carotenoids in white clover and common sainfoin was less than half of the content measured in red clover and alfalfa. The discrepancy stems from the character of particular plant species and the particular soil and climatic conditions, as well as stage of the respective plant’s development or the age of the perennial plant. The concentration of carotenoids measured in black salsify leaves in plants grown from one-year-old seeds was twice as high as in plants grown from four-year-old seeds. This likely indicates that seeds may not have fully matured before the harvest or that they aged in storage, which in turn may have impacted their biochemical composition [37]. Seeds contain gibberellins which are active growth stimulators. They are involved mainly in the process of seed formation, but are significant to the physiological activity of a seed as a whole. Growth inhibitors can arrest seed metabolism. The growth and development of plants is largely regulated by a variety of hormones which play a key role in physiological processes at every stage of a plant’s life [53]. The highest content of chlorophyll *a* and *b* in the year the plants were sown and in the phase of alfalfa’s full budding was, respectively, 2061 µg∙g^− 1^ and 645 µg∙g^− 1^, while the content of carotenoids was lower at 217 µg∙g^− 1^ [54]. In our present study, Chl *a* was over 50% lower in alfalfa leaves during the leaf development phase, and Chl *b* was as much as 60% lower. The content of carotenoids was 28% lower. Legumes growing in the shade contained more chlorophyll pigment when compared to plants growing in full sunlight [26]. In other studies, it was observed that the content of chlorophyll decreased under salt stress conditions, which reduced the rate of photosynthesis in *Medicago truncatula* plants [55]. The concentration of chlorophyll *a*, chlorophyll *b*, and carotenoids in the leaves of *Sulla carnosa*, a fodder legume, increased by, respectively, 93%, 36%, and over 300% in nitrogen-rich and high salination conditions [29]. In our study, the content of Chl *a*, Chl *b*, and carotenoids, was the lowest in common sainfoin and comparatively 213%, 244%, and 243% higher in red clover, white clover, and alfalfa, respectively. At the same time in another study, despite reduced availability of phosphorus, the photosynthetic efficiency remained practically unchanged when measured in legumes originating from the Mediterranean Fynbos ecosystem [56].

**Table 3:** Photosynthetic pigments in perennial legumes

Plant species	Chl a (µg. g^− 1^)	Chl b (µg. g^− 1^)	Car (µg. g^− 1^)	Chl a + b (µg. g^− 1^)
White clover	926.18^ab^ ± 196.41	389.86^a^ ± 53.31	56.94^a^ ± 25.33	1316.03^ab^ ± 243,16
Red clover	1186.08^a^ ± 192.95	351.54^ab^ ± 53.82	143.62^a^ ± 52.21	1537.62^a^ ± 246.76
Alfalfa	882.63^ab^ ± 144.89	248.34^b^ ± 35.17	157.21^a^ ± 45.18	1130.98^ab^ ± 177.05
Common sainfoin	557.35^b^ ± 35.76	159.94^b^ ± 8.99	64.57^a^ ± 29.55	717.30^b^ ± 44.75

Fisher Test (LSD) α < 0.05, ± standard deviation, a–b– different letters indicate statistically significant differences between the plants, *n* = 3

Chl *a*– chlorophyll *a*, Chl *b*– chlorophyll *b*, Car– carotenoids, Chl *a + b*– chlorophyll *a + b*

**Fig. 4:**
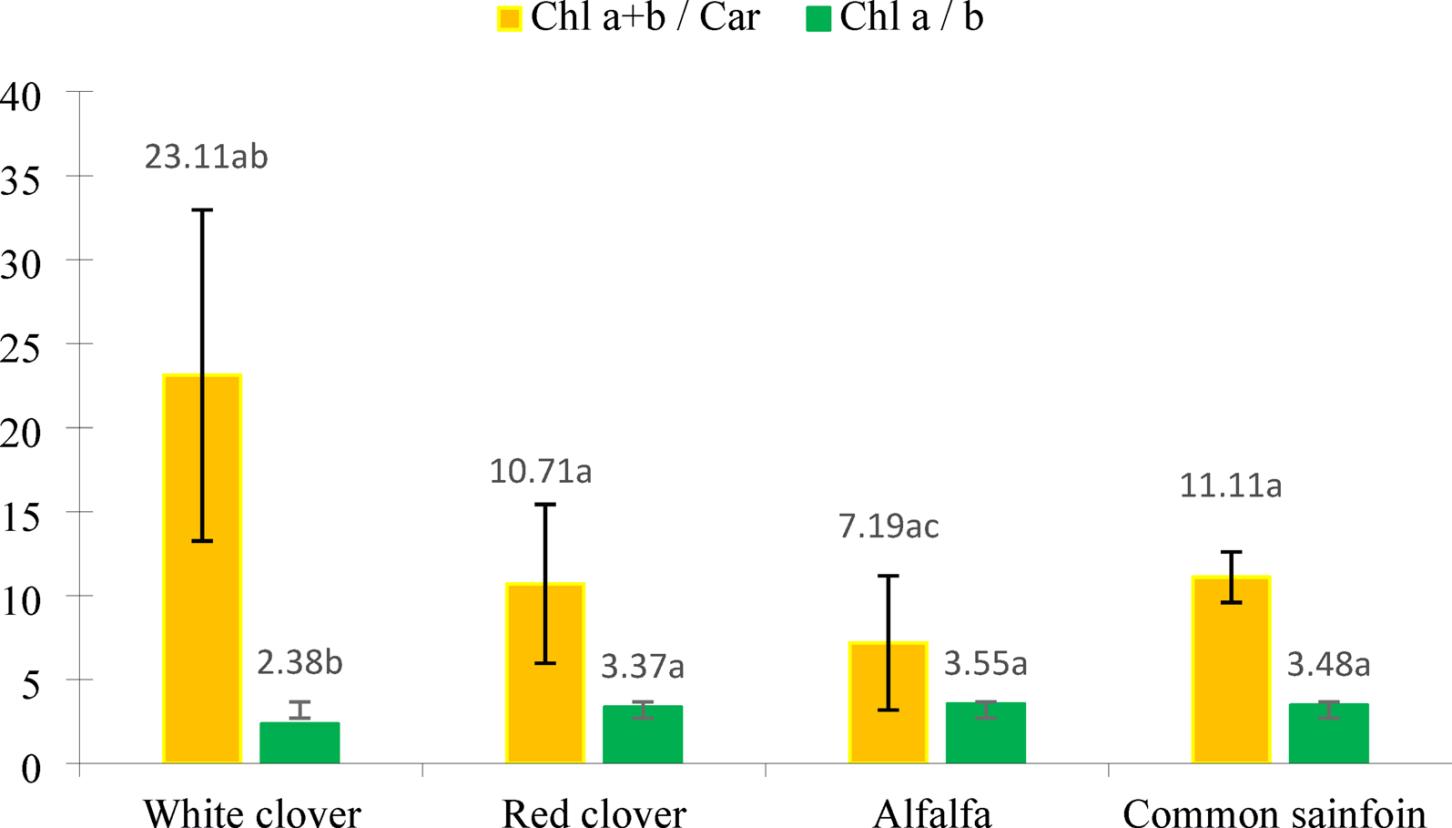
Mean values of the ratios of chlorophyll *a + b* to carotenoids, and chlorophyll *a* to *b*, ± standard deviation, a–c– different letters indicate statistically significant differences between the plants

In plants, the contents of individual pigments (chlorophylls and carotenoids) and their ratio change under stress or aging of leaves [57]. Chlorophylls a and b are usually present in plants in a 3:1 ratio [13, 36, 58, 59]. In plants growing under shaded conditions, this ratio ranges from 2.0 to 2.8, while in plants developing in full sunlight it ranges from 3.5 to 4.9 [60]. The mean content of chlorophyll a has been reported to be three times higher than the corresponding content of chlorophyll b [13, 36]. In our study, in three of the plant species selected, Chl *a* also exceeded Chl *b* over threefold (Fig. 4.). The only exception was white clover, where the content of chlorophyll *a* was only two times higher relative to chlorophyll *b*. The difference observed between white clover and the other species was statistically significant at α < 0.05. In turn, the Chl *a + b* / Car ratio was within the range from 7.19 (alfalfa) to 23.11 (white clover) (Fig. 4.). Lower values of Chla/b and higher values of Chl *a + b* / Car are characteristic of plants growing in shaded conditions as compared to ones growing in full sunlight, reflecting differences in the composition of photosynthetic antennae and leaf morphology [61, 62].

**Fig. 5:**
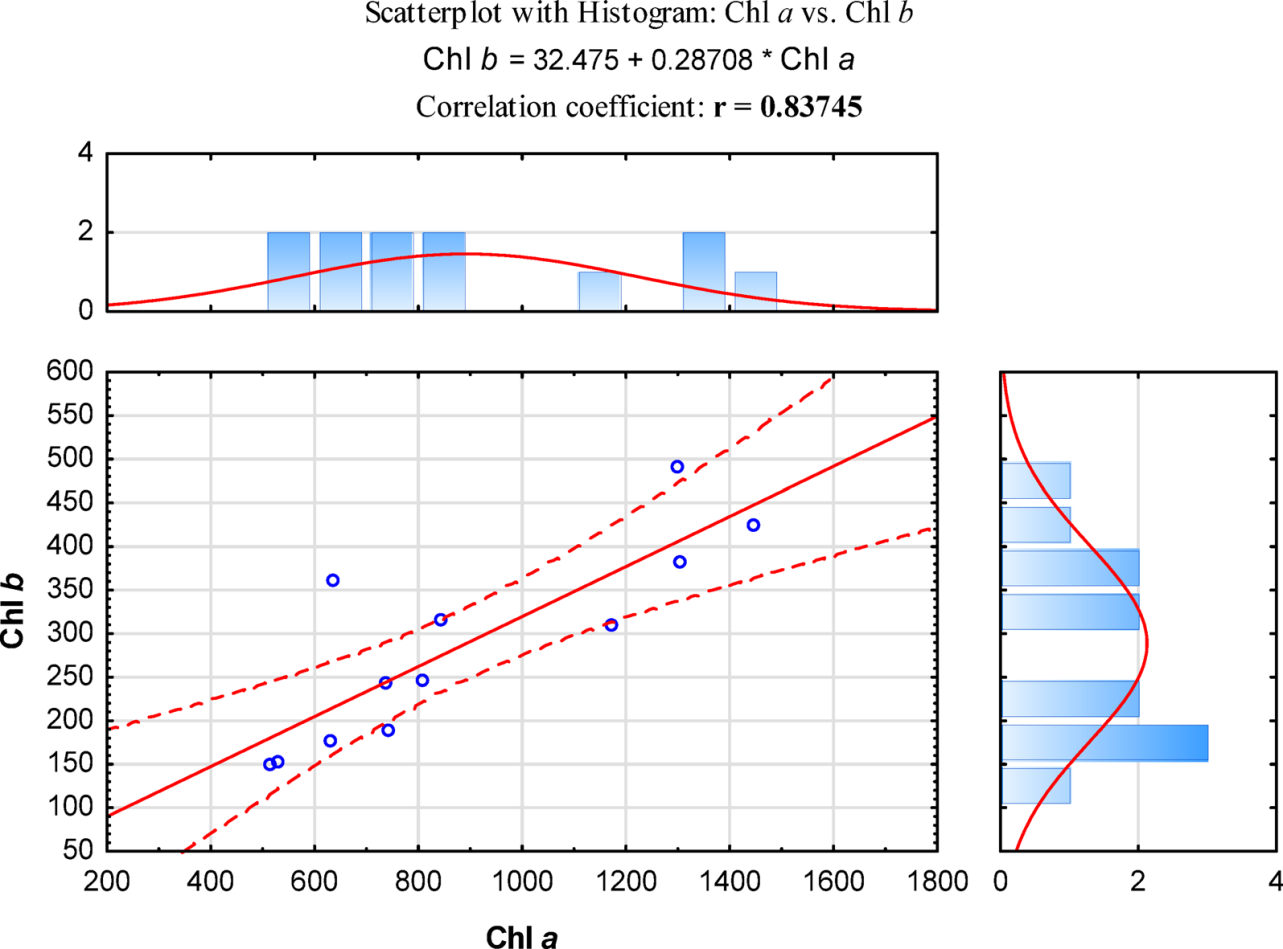
Scattering plot for all the plant species studied with histogram for the correlation between chlorophyll *a* and chlorophyll *b*. The bold value of the correlation coefficient r indicates a significant statistical difference, at a significance level of α < 0.05

Moreover, a strong positive correlation was observed between the content of chlorophylls *a* and *b* (Fig. 5.). The positive correlation between the variables is statistically significant at the α < 0.05 level indicating that an increase in one variable is simultaneously associated with an increase in the other.

## Conclusions

The studied legumes differed significantly in terms of their photosynthetic efficiency parameters, Chl *a* fluorescence lifetimes, and content of photosynthetic pigments in the leaves, despite belonging to the same plant family. This suggests that environmental conditions have a significant impact on the photosynthetic parameters of respective plants.

The observed photosynthetic efficiency was the highest in common sainfoin and only slightly lower, by approx. 3–5%, in alfalfa and white clover. In turn, red clover was found to have the highest content of chlorophyll *a* and *a + b* as well as the longest Chl *a* fluorescence lifetime when compared to the other Fabaceae. It was observed that fluorescence lifetimes decreased with decreasing content of chlorophyll *a* in the leaves for red clover, white clover, and sainfoin, with alfalfa being the only exception.

The measurements of chlorophyll fluorescence conducted in vivo, which provided the basis for the determination of Y(II) and ETR, alongside measurements of chlorophyll fluorescence lifetimes, provided information on the plants’ response to the light conditions during the measurements, while the pigment content in the leaves reflected the plants’ adaptation, including in terms of the size of photosynthetic antennae. Various adverse environmental conditions (draught, excessive sunlight, cold, frost, etc.) can disrupt plant growth and development processes. Crops grown in natural conditions are particularly susceptible to such environmental influence. Methods utilizing the phenomenon of chlorophyll fluorescence are particularly useful in monitoring crops and ecosystems exposed to phytotoxic hazards. Given their non-invasiveness, speed and ease of use, as well as their increasing availability, such solutions provide a convenient method for early detection of changes affecting plants, in particular with regard to their photosynthetic efficiency, and determination of the overall physiological condition of crops exposed to adverse environmental conditions.

Future research should pay particular attention to long-term observations of the variability of photosynthetic activity in perennial plants at different stages of their development. An in-depth analysis of the adaptability to changing environmental conditions, such as fluctuations in temperature and precipitation, could enable more comprehensive conclusions to be formulated about the mechanisms of the physiological response of plants to environmental stress.

## Supplementary Information

Below is the link to the electronic supplementary material.

Supplementary Material 1

## Data Availability

Data will be available on request. Correspondence and requests for materials should be addressed to A.D.-H.
