# Supplementary material for: A case study in photosynthetic parameters of perennial plants growing in natural conditions
Source: BMC Plant Biol. 2025 Aug 8;25:1044. doi: 10.1186/s12870-025-07133-1 (PMC12333072; doi:10.1186/s12870-025-07133-1)
Supplement: Supplementary file 1 — Supplementary Material 1 [file 12870_2025_7133_MOESM1_ESM.docx]

| **Table S1**. Photosynthetic parameters measured for perennial leguminous plants | | | | | | |
| --- | --- | --- | --- | --- | --- | --- |
| **PLANT SPECIES** | **PAR**  **(µmol m^-2^ s^-1^)** | **TEMP. (^o^C)** | **F** | **Fm^’^** | **Y (II)** | **ETR**  **(µmol electrons m⁻² s⁻¹)** |
| White clover | 65.50^d^  ±27.34 | 23.60^ac^  ±0.36 | 441.60^bc^  ±87.18 | 1432.30^a^  ±182.48 | 0.692^a^  ±0.039 | 19.04^d^  ±7.90 |
| Red clover | 915.8^a^  ±349.09 | 24.03^a^  ± 0.30 | 627.20^a^  ±205.91 | 1344.90^a^  ±391.48 | 0.521^b^  ±0.113 | 187.74^a^  ±56.16 |
| Alfalfa | 560.60^b^  ±382.92 | 23.06^b^  ±0.71 | 520.10^ac^  ±102.10 | 1389.6^a^  ±164.69 | 0.618^a^  ±0.103 | 133.10^b^  ±63.32 |
| Common sainfoin | 271.10^cd^  ±222.96 | 23.25^bc^  ±0.72 | 406.60^bc^  ±49.18 | 1470.00^a^  ±383.28 | 0.697^a^  ±0.125 | 75.12^c^  ±59.15 |
| Fisher Test (LSD) α<0.05, ± standard deviation, a-d – different letters indicate statistically significant differences between the plants, n=10.  F – fluorescence intensity, measured in light adapted state before the saturating pulse, Fm’ – fluorescence intensity reached during the saturating pulse in light adapted state, PAR – photosynthetically active radiation, ETR – relative electron transport rate, Y (II) – effective quantum yield of photochemical energy conversion at PS II reaction centers, TEMP. – temperature of the leaf during measurement. | | | | | | |

**Figure S2.** Scattering plot for all the plant species studied with a histogram showing correlations between: (**A**) photosynthetically active radiation (PAR) and relative electron transport rate (ETR); (**B)** quantum efficiency of the photochemical reaction Y(II) and relative electron transport rate (ETR). The bold value of the correlation coefficient r indicates a significant statistical difference, at a significance level of α < 0.05.
